# Utility of a 21-gauge Menghini-type biopsy needle with the rolling method for an endoscopic ultrasound-guided histological diagnosis of autoimmune pancreatitis: a retrospective study

**DOI:** 10.1186/s12876-020-01590-8

**Published:** 2021-01-07

**Authors:** Koichiro Tsutsumi, Toru Ueki, Yasuhiro Noma, Kunihiro Omonishi, Kyotaro Ohno, Soichiro Kawahara, Takashi Oda, Hironari Kato, Hiroyuki Okada

**Affiliations:** 1 Departments of Internal Medicine, Fukuyama City Hospital, 5-23-1, Zao-cho, Fukuyama-City, Hiroshima 721-8511 Japan; 2 Departments of Internal Medicine and Pathology, Fukuyama City Hospital, 5-23-1, Zao-cho, Fukuyama-City, Hiroshima 721-8511 Japan; 3 Department of Gastroenterology and Hepatology, Okayama University Hospital, 2-5-1, Shikata-cho, Kita-ku, Okayama-City, Okayama 700-8558 Japan; 4 Department of Internal Medicine, National Hospital Organization Fukuyama Medical Center, 4-14-17, Okinogami-cho, Fukuyama-City, Hiroshima 720-8520 Japan

**Keywords:** EUS-FNB, ICDC, Sample area, Good-quality tissue

## Abstract

**Background:**

The histological diagnosis of autoimmune pancreatitis (AIP) by an endoscopic ultrasound (EUS)-guided approach is still challenging.

**Methods:**

We investigated the utility of the 21-gauge Menghini-type biopsy needle with the rolling method for the histological diagnosis of AIP, in comparison with conventional 22-gauge needles. Among total 28 patients, rate of definitive histological diagnosis, acquired sample area of tissue, rate of histopathological diagnosis of AIP, and adverse events were retrospectively analyzed.

**Results:**

Definitive histological diagnoses were successfully accomplished in all 14 patients (100%) treated with a Menghini-type needle, and in 57% of cases (8/14) treated with conventional 22-gauge needles (*P* < 0.001). The median sample area of the tissue, except for blood contamination, was remarkably larger by the Menghini-type needle than by conventional-type needles (6.2 [IQR, 4.5–8.8] versus 0.7 [IQR, 0.2–2.0] mm^2^, *P* < 0.001), and the area per punctures was approximately 4 times larger (1.4 [IQR: 0.9–2.9] versus 0.3 [IQR: 0.1–0.6] mm^2^/puncture, *P* < 0.001). Based on the International Consensus Diagnostic Criteria, lymphoplasmacytic infiltration, abundant IgG4-postive cells, storiform fibrosis, and obliterative phlebitis were found in 86%/29%, 64%/0%, 36%/0%, and 7%/0% patients who were treated with the Menghini-type needle and conventional-type needles, respectively. Consequently, histopathological diagnosis with type 1 AIP (lever 1 or 2) was achieved in 9 patients (64%) treated with the Menghini-type needle and in no patient treated with conventional-type needles (*P* < 0.001). Two patients who had mild post-procedural pancreatitis improved with conservative treatment, and no bleeding occurred in patients treated with the Menghini-type needle.

**Conclusion:**

EUS-guided rolling method with the 21-gauge Menghini-type biopsy needle is useful for the histopathological diagnosis of AIP, due to its abundant acquisition of good-quality tissue from the pancreas.

## Background

Autoimmune pancreatitis (AIP) is an unusual type of pancreatitis, diagnosed using five criteria (parenchymal imaging, ductal imaging, serology, histology of the pancreas, and response to steroids) according to the International Consensus Diagnostic Criteria (ICDC) [1, 2]. In clinical practice, the definitive diagnosis of AIP is sometimes difficult due to a lack of diagnostic evidence based on noninvasive pancreatic imaging and serology, especially for patients with segmental or focal pancreatic enlargement. Therefore, histological findings are crucial for the diagnosis of type 1 AIP, whose defining feature is lymphoplasmacytic sclerosing pancreatitis (LPSP), as well as type 2 AIP, whose defining feature is idiopathic duct-centric pancreatitis (IDCP). However, the ICDC states that histological assessments should be performed using only surgical or core biopsy specimens, which require an invasive procedure [3].

Endoscopic ultrasound-guided fine-needle aspiration (EUS-FNA) is standardly performed for the diagnosis of solid pancreatic masses [4–6], and the advent of a dedicated needle has improved the rate of sufficient tissue sampling [7–10]. Several reports have found that an EUS-guided fine-needle biopsy (EUS-FNB) and EUS-FNA using a 19-gauge trucut needle and 19- and 22-gauge conventional FNA or FNB needles were feasible and safe for obtaining pancreatic tissue specimens for the diagnosis of AIP [11–21]. Recently, Notohara et al. reported an extremely important point that EUS-FNB with large tissue amounts was useful for diagnosing type 1 AIP, notably by facilitating successful IgG4 immunostaining [22]. From that perspective, further improvement is still needed in order to obtain greater good-quality tissue samples for evaluating distinctive histological findings of AIP.

The 21-gauge Menghini-type biopsy needle is a newly developed needle for EUS-FNB that was recently reported to provide high-quality specimens for histological evaluations for the diagnosis of solid pancreatic masses, especially in terms of both sample cellularity and blood contamination, compared with a 22-gauge conventional needle [23, 24]. The two special features of this needle, namely the tapered beveled-edge of the outer needle and the inner needle connected to a barrel equipped with an aspiration piston, can substantially improve the acquisition of ample tissue with little blood contamination. However, in the field of liver biopsies, a unique technique has long been applied wherein the needle is quickly punctured into the target lesion, turned around once or twice and then removed in order to wrench out core tissue [25]. This technique is referred to as the “rolling method” in the present study. In our ex vivo test, the Menghini-type needle was found to be the easiest of the needles we evaluated to rotate through the channel of the endoscope. Therefore, we hypothesized that the combination of these strengths would improve the histological diagnostic ability of AIP.

In the present study, we clarified the utility of this 21-gauge Menghini-type biopsy needle combined with the “rolling method” for the histological diagnosis of AIP.

## Methods

### Study design

This was a retrospective, single-center study conducted at Fukuyama City Hospital in Japan. The study was performed under the approval of the ethics committees of the hospital.

### Patients

Between January 2010 and November 2018, 49 patients were ultimately diagnosed with AIP according to the ICDC. Among them, cytological and histological assessments by EUS-FNA and EUS-FNB were performed in 41 patients.

During the early period (January 2010 to September 2015), several different types of conventional FNA or FNB needles, such as the EchoTip ultra (COOK Japan, Tokyo, Japan), Expect (Boston Scientific Japan, Tokyo, Japan), SonoTip Procontrol (Medi-Globe GmbH, Rosenheim, Germany), and EchoTip ProCore (COOK), were used. In the later period (October 2015 to November 2018), a 21-gauge Menghini-type needle (EUS Sonopsy CY; Hakko Medical, Nagano, Japan) and a 22-gauge Franseen-type needle (Acquire; Boston Scientific) were used for these procedures. The selection of these devices for use was dependent on the period and the endoscopists’ preference.

Of note, 5 patients treated using 25-gauge conventional FNA needles were excluded due to low likelihood of acquiring adequate tissue, and 5 patients treated with the Franseen-type needle, 2 treated with the EchoTip ProCore and 1 treated with the 19-gauge needle were excluded due to the small number of patients. Therefore, a total of 28 patients were included in this study: the 14 patients treated with the 21-gauge Menghini-type needle and the 14 patients treated with 22-gauge conventional needles as a historical control.

### EUS-FNA and EUS-FNB procedures

A linear echoendoscope (UCT-260; Olympus, Tokyo, Japan) was used for EUS-FNA and EUS-FNB. These procedures were performed by mainly two experienced endoscopists under conscious sedation with midazolam and pethidine.

For patients with diffuse pancreatic enlargement, puncture was performed mainly via the transgastric route because of its technical ease [26, 27]. For patients with segmental or focal pancreatic enlargement, puncture was performed via the transgastric or transduodenal route, depending on the location.

**Fig. 1:**
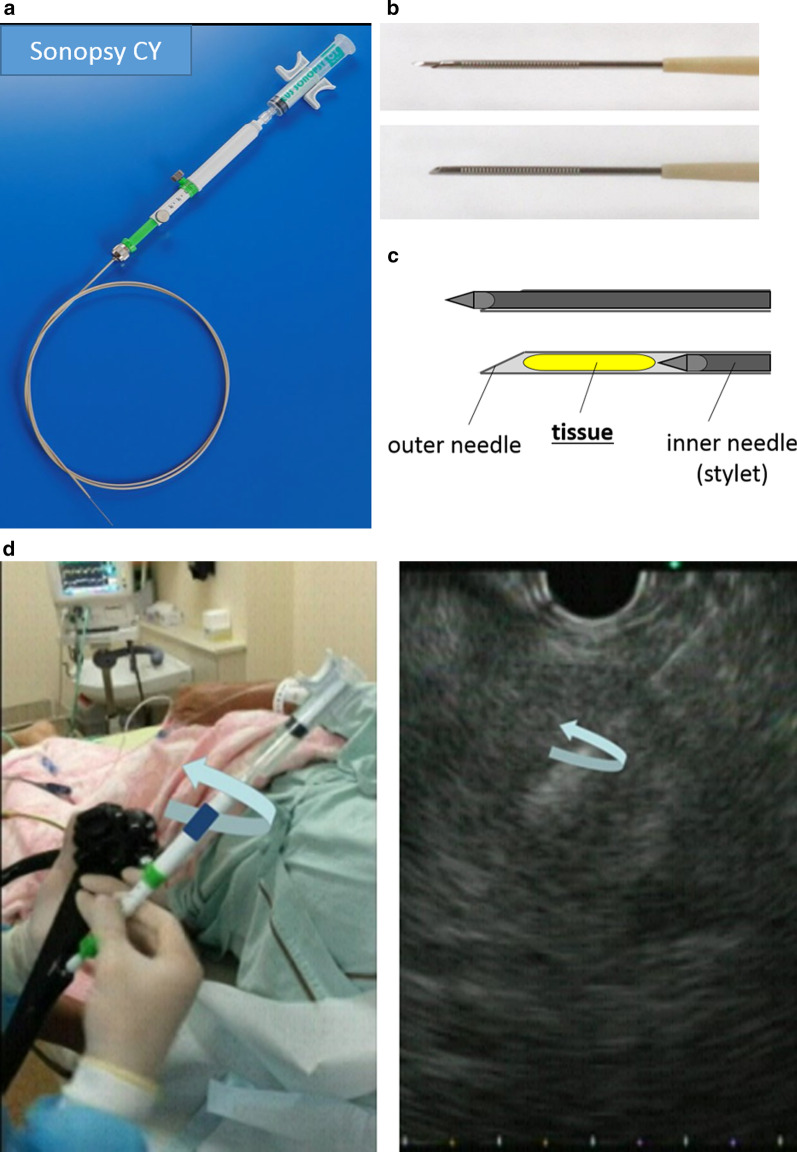
A 21-gauge Menghini-type needle. **a** Whole image of the 21-gauge endoscopic ultrasound-guided fine needle aspiration biopsy needle (EUS Sonopsy CY). **b**, **c** Image of the needle tip before (upper) and after (lower) aspiration using the piston, respectively. **d** After puncture with aspiration under 10 mL of negative pressure, the handle of the needle was turned until the tip of the needle was confirmed to be rotated in the lesion. To reduce the contamination of blood, this procedure was repeated only three times, and the needle was removed

Figure 1 shows the 21-gauge Menghini-type needle (EUS Sonopsy CY), which was originally made for performing liver biopsies (Sonopsy C1; HAKKO Medical) [23, 24, 28]. This needle has two features of note. First, the tapered shape of the beveled-edge can facilitate obtaining adequate core tissue with just a few strokes. Second, unlike other FNA and FNB needles, keeping the inner needle attached to the plunger of the syringe within the outer needle during aspiration helps obtain high-quality tissue without crushing. After positioning at the intended puncture site, the outer barrel was quickly moved forward until the puncture needle was slightly inserted into the intended site. For aspiration, the piston of a 10-ml syringe was drawn until it was locked without removal of the inner needle (stylet). Following aspiration for at least 3 s to allow negative pressure to accumulate at the tip, the needle was pushed forward through the intended site, and then, in order to wrench out core tissue, we additionally turned the outer barrel until the tip of the needle was confirmed to have been rotated simultaneously within the lesion in the EUS view, just as in the liver biopsy technique [25] known as the “rolling method”. This procedure was repeated only two or three times in order to reduce the risk of contamination with blood induced by performing too many strokes, and then the needle was removed.

As the conventional EUS-FNA, the 22-gauge needles were punctured at the target lesion, and the stylets were removed. The needles were then moved back and forth within the lesion with negative pressure 15–20 times per puncture before being removed. In addition, the fanning technique was used for all 28 patients.

After performing EUS-FNA and EUS-FNB, endoscopic retrograde pancreatocholangiography (ERCP) was basically performed in a single-session [29–31] to achieve pancreatographic imaging and biliary drainage if patients had obstructive jaundice.

### On-site evaluations

All samples were first assessed for cellular adequacy by cytopathologists using Papanicolau staining. After adequate cellularity for cytology had been confirmed, the remaining and additional samples were preserved for histological evaluations. At that time, endoscopists tried to identify whitish core tissue on a glass slide, which was so-called “MOSE” (macroscopic on-site quality evaluation) [32], and the procedure was performed up to six punctures.

### Pathological evaluations

Two pathologists, who blinded to the type of needle, performed cytological and histological evaluation. A benign or malignant status was cytologically determined. As a histological evaluation, formalin-fixed paraffin-embedded tissue was stained with hematoxylin–eosin (H&E) and IgG4. In addition, obliterative phlebitis (OP) was diagnosed by Elastica van Gieson staining. According to the ICDC, the LPSP findings (marked lymphoplasmacytic infiltration, OP, storiform fibrosis [SF], abundant IgG4-positive plasma cells [> 10 cells/high-power field {HPF}]) and the IDCP findings (granulocytic infiltration of duct wall [GEL], and absent or scant IgG4-positive cells) were evaluated. Based on these findings, level 1 and 2 criteria were adopted for the diagnosis of AIP.

In addition, the total area of the acquired tissue, except for mucus or blood contamination, was accurately measured under a photomicroscope (microscope, BX53, Olympus, Tokyo, Japan; camera, DP73, Olympus) using imaging software (cellSens, standard 1.8.1, Olympus).

### Study outcomes

As a primary outcome, success rate of confirming histological diagnosis, such as AIP and pancreatitis due to chronic inflammation, was evaluated. As secondary outcomes, the amount of the acquired tissue, the histological findings, cytological diagnosis, and adverse events were also assessed. The amount of the acquired tissue was defined as the total area of the acquired tissue measured by the imaging software, as described above. Histological findings and cytological diagnosis were assessed, as described in the *Pathological evaluations*. Adverse events and their severity related to the procedures were defined and graded according to ASGE lexicon [33].

### Statistical analyses

Continuous data are presented as medians with the range or interquartile range (IQR). Continuous variables and frequency distribution were compared with the Mann–Whitney U test and Fisher’s exact or χ^2^ test, respectively. A *P*-value < 0.05 was considered statistically significant. All statistical analyses were performed with the GraphPad Prism software program, ver. 6.0 (GraphPad Software, San Diego, CA, USA).

## Results

### Patients’ characteristics

Table 1 summarizes the characteristics of the 28 included patients. In this study, 14 patients each were managed using a Menghini-type needle with the rolling method and conventional-type needles. The ratio of male-to-female patients was approximately 4:1 in both groups. Based on the ICDC, the diagnosis of definitive type 1 AIP was obtainable in 23 patients (82%) but no patients had type 2 AIP before histological evaluation. No pancreatic lesions developed malignancy during over 3-years follow-up.

**Table 1:** Patients' characteristics and clinical findings

	Menghini-type	Conventional-type	*P* value
	(N = 14)	(N = 14)	
Male (n [%])	11 (71)	12 (86)	0.622
Age (years, median [range])	71 (50–79)	62 (54–75)	0.018
Obstructive jaundice (n [%])	4 (29)	4 (29)	1.000
HbA1c (%, median [range])	7.3 (5.2–11.7)	5.8 (5.1–8.7)	0.032
Diagnosis using the ICDC without histological findings of EUS-guided approach (n [%])			
Definitive type 1 AIP	13 (93)	10 (71)	0.139
Not definitive type 1 AIP	1 (7)	4 (29)	
Type 2 AIP	0	0	
Median follow-up period (days [range])	1116 (297–3303)	1747(683–3303)	0.028
Pancreatic imaging			
Enlargement with delayed enhancement			
Diffuse/Segmental or Focal	11/3	7/7	0.052
MPD narrowing			
Long (≥ 1/3) or multiple/Segmental or Focal	11/2	6/4	0.115
Location of the enlarged part			0.412
Whole	8 (57)	7 (50)	
Head	1 (7)	1 (7)	
Body	0	1 (7)	
Tail	0	2 (14)	
Head and body	0	1 (7)	
Body and tail	3 (21)	1 (7)	
Head and tail	2 (14)	1 (7)	
Serology			
IgG4 (mg/dl, median [range])	364.3 (104–1090)	267 (32–1270)	0.828
IgG4 ≥ 135 mg/dl (N [%])	11 (79)	11 (79)	1.000
Level 1, ≥ 270 mg/dl	9 (64)	7 (50)	
Level 2, ≥ 135 mg/dl but < 270 mg/dl	2 (14)	4 (29)	
Other organ involvement			
Sclerosing cholangitis	5 (36)	6 (43)	0.699
Retroperitoneal fibrosis	4 (29)	3 (21)	0.663
IgG4-related kidney disease	3 (21)	0	0.067
Sialadenitis	2 (14)	0	0.142
Dacryoadenitis	1 (7)	0	0.309
Inflammatory bowel disease	0	0	–
Response to steroids (n [%])	14 (100)	11^a^ (100)	–

*ICDC* International Consensus Diagnostic Criteria, *AIP* Autoimmune pancreatitis, *MPD* Main pancreatic duct

^a^Remaining three patients improved before introduction of treatment with steroids

### Clinical findings of the enrolled patients

As shown in Table 1, segmental or focal enlargement of the pancreas was more common in patients treated with conventional-type needles than in those treated with Menghini-type needles (n = 7 [50%] and n = 3 [21%], respectively; not significant). However, a focal lesion of the pancreas head alone, which required a transduodenal approach for EUS-FNB, was detected in 1 (7%) in both groups. Elevated serum IgG4 levels (≥ 135 mg/dl) were seen in 75% of patients in both groups. Steroid administration was conducted in 25 patients (89%) and was effective in all, while the remaining 3 patients showed improvement of pancreatic enlargement without steroid therapy within 6 months.

### Success rate of confirming histological diagnosis and acquired tissue area by EUS-FNB

Definitive histological diagnoses were successfully achieved in all patients treated with a Menghini-type biopsy needle, with a median of 4 (IQR: 3–5) punctures (Table 2). In contrast, conventional-type needles obtained the diagnosis in 57% of cases (8/14), with a median of 3 (IQR: 1–4) punctures.

**Table 2:** Results Associated with EUS-FNA and EUS-FNB

	Menghini-type 21-gauge	Conventional-type 22-gauge	*P* value
	N = 14	N = 14	
Needles	Sonopsy 14	Expect 7	
		Sonotip 4	
		EchoTip 3	
Route for puncture			0.231
Transgastric	12 (86)	8 (57)	
Transduodenal	1 (7)	4 (29)	
Transgastric and transduodenal	1 (7)	2 (14)	
Number of punctures (median [IQR])	4 (3–5)	3 (1–4)	0.016
Stroke length of needles (median [IQR])	1.6 (1.5–2.0)	1.5 (1.5–1.9)	0.664
Successful confirmation of histological diagnosis (n [%])	14 (100)	8 (57)	0.006
A confirmed cytological diagnosis of no malignancy (n [%])	14 (100)	12 (86)	0.142
Adverse events (n [%])	2 (14)	0	0.142
Histological findings			
Periductal lymphoplasmacytic infiltrate without granulocytic infiltration	12 (86)	4 (29)	0.002
Obliterative phlebitis	1 (7)	0	0.310
Storiform fibrosis	5 (36)	0	0.014
Abundant (> 10 cells/HPF) IgG4-positive cells	9 (64)	0	< 0.001
Histological diagnosis for AIP			< 0.001
Level 1	5 (36)	0	
Level 2	4 (29)	0	

*IQR* interquartile range, *HPF* high-power field

**Fig. 2:**
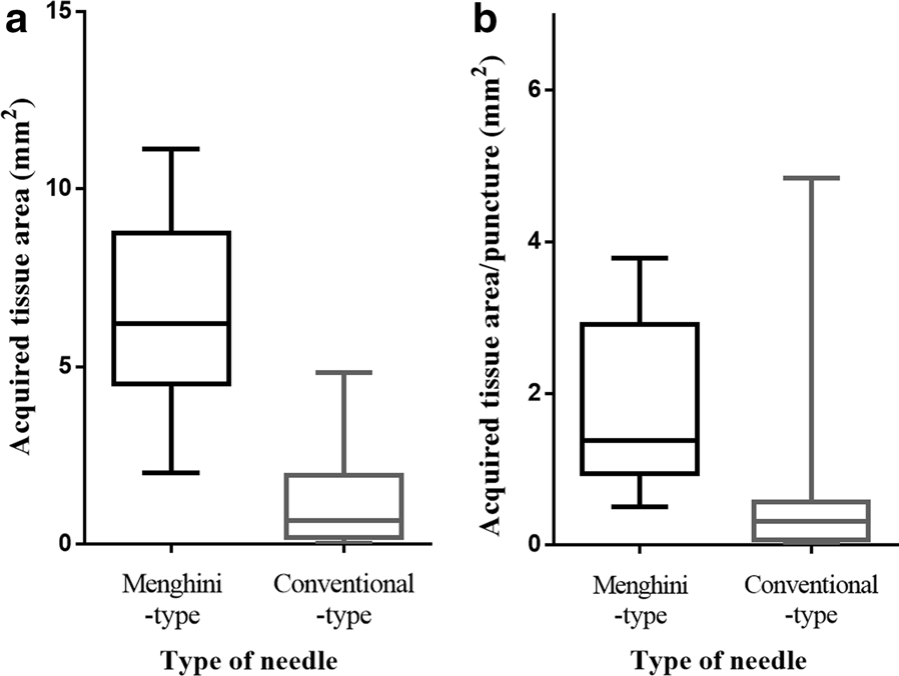
Comparison of the amount of acquired tissue area measured by imaging software “cellSens®”. **a** The median acquired sample area of the tissue, except for that with blood contamination, was markedly larger with the Menghini-type needle than with conventional-type needles (6.2 [IQR, 4.5–8.8] versus 0.7 [IQR, 0.2–2.0] mm^2^, *P* < 0.001). **b** The median sample area per punctures was approximately 4 times larger (1.4 [IQR: 0.9–2.9] versus 0.3 [IQR: 0.1–0.6] mm^2^/puncture, *P* < 0.001)

**Fig. 3:**
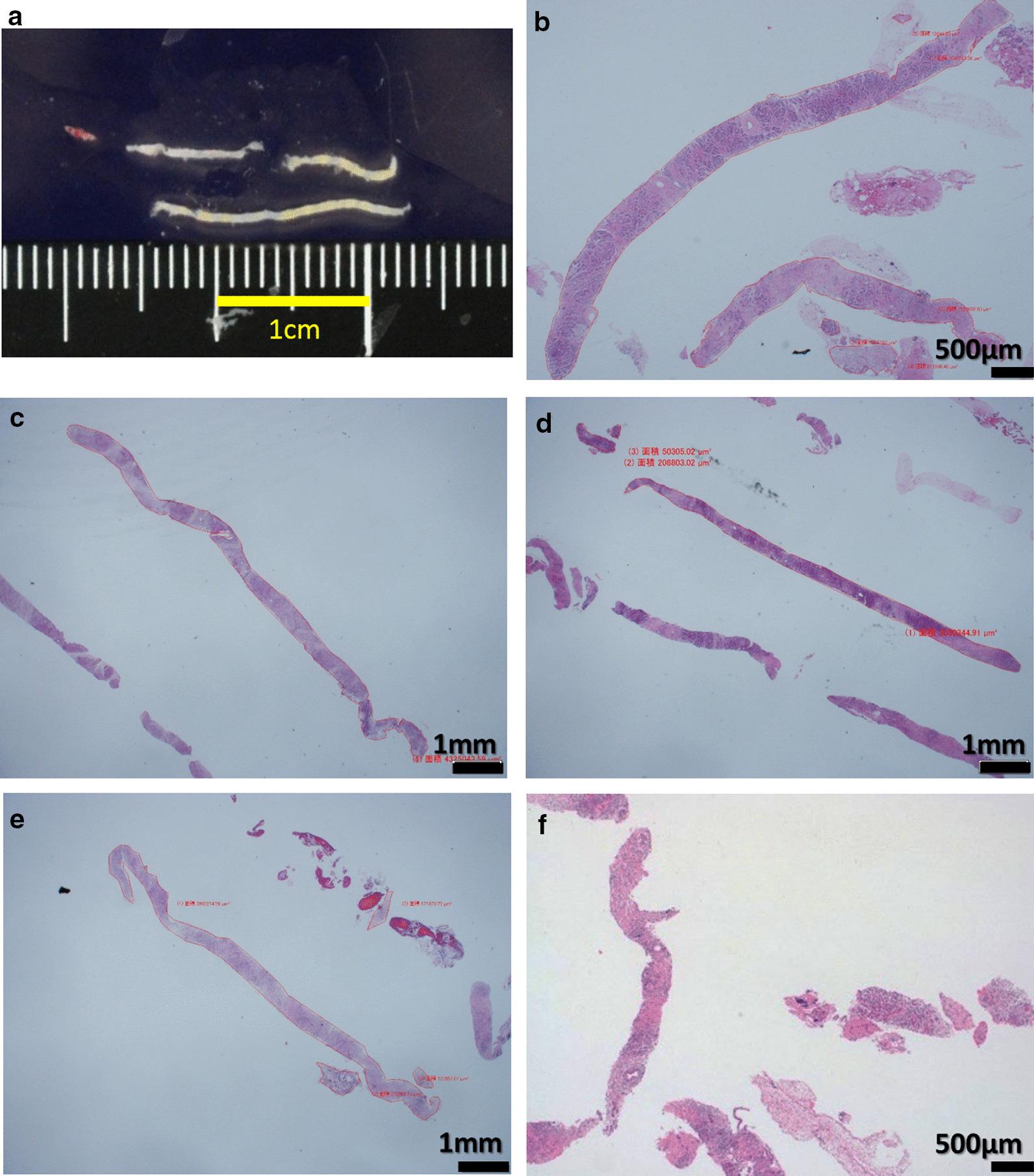
Pancreatic tissue obtained by the 21-gauge Menghini-type needle with the rolling method. **a** A macroscopic image shows abundant whitish tissue with little blood contamination. **b**–**f** Microscopic images of the tissue with hematoxylin–Eosin (HE) staining are shown among five representative cases. Exceedingly long, high-quality pancreatic tissues can be acquired by this procedure

**Fig. 4:**
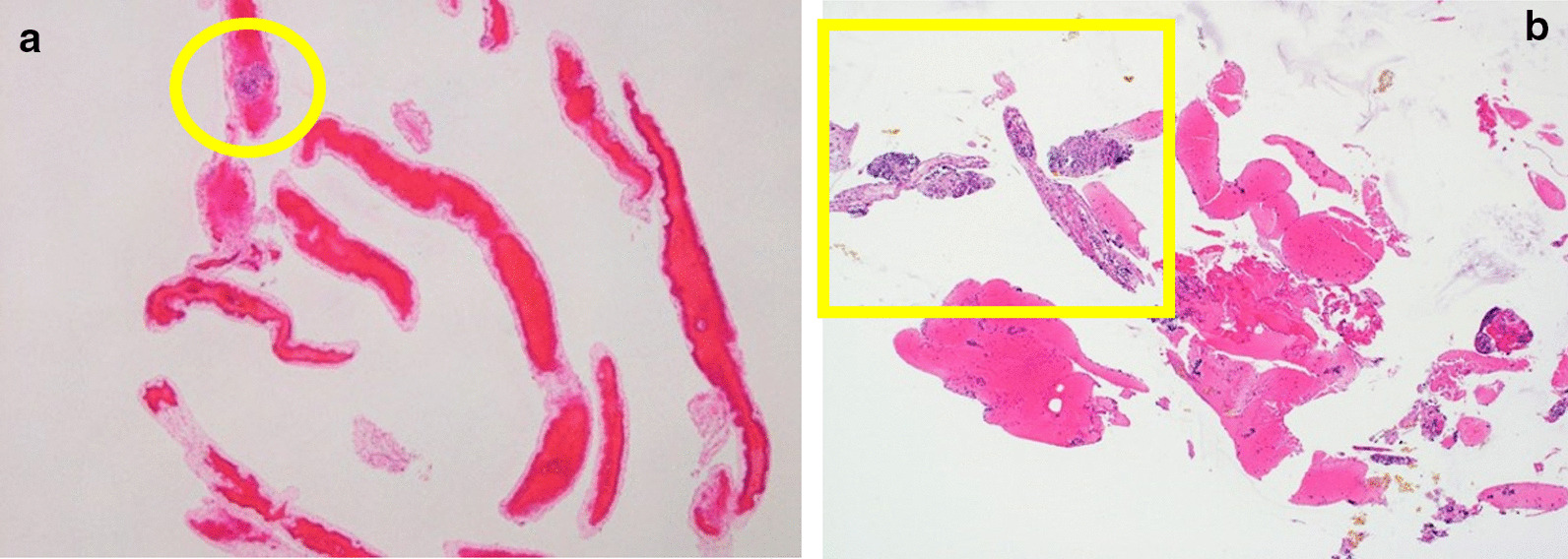
Pancreatic tissue obtained by a conventional 22-gauge needle. **a** A microscopic image of Hematoxylin–Eosin (HE) staining shows a rather small amount of tissue with substantial fibrin and neutrophils. This tissue proved insufficient for a histological evaluation (yellow ellipse). This case was identical to that in Fig. 3b. **b** A microscopic image of HE staining shows a rather small amount of tissue with substantial blood. This tissue proved insufficient for a histological evaluation (yellow rectangle). This case was identical to that in Fig. 3c

Regarding the sample area of the acquired tissue, the median sample area was significantly larger by a Menghini-type needle than by conventional-type needles (6.2 [IQR, 4.5–8.8] versus 0.7 [IQR, 0.2–2.0] mm^2^, *P* < 0.001) (Fig. 2, 3, 4). In addition, the area per punctures was approximately 4 times larger by a Menghini-type needle than by conventional-type needles (1.4 [IQR: 0.9–2.9] versus 0.3 [IQR: 0.1–0.6] mm^2^/puncture, *P* < 0.001). In contrast, the stroke lengths of the needles were not significantly different between the two approaches. Taken together, these findings show that using the 21-gauge Menghini-type needle with the rolling method was a considerably superior approach to using conventional 22-gauge needles for histological evaluations, resulting in obtaining abundant tissue with little blood contamination.

### Histological findings by EUS-FNB

**Fig. 5:**
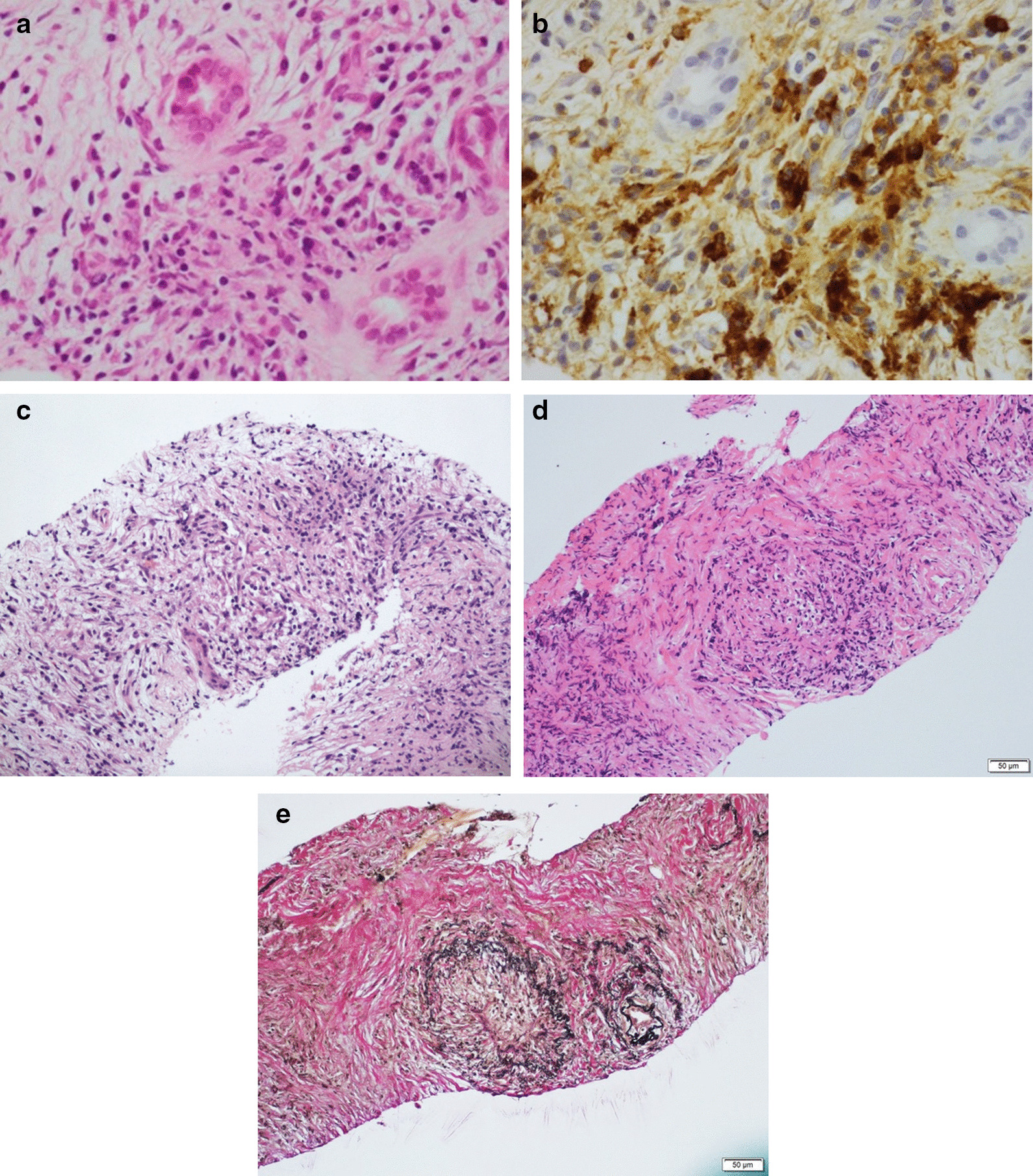
Histological findings for the diagnosis of AIP acquired by the 21-gauge Menghini-type needle with the rolling method. **a** Hematoxylin–Eosin (HE) staining shows marked lymphoplasmacytic infiltration and fibrosis in high-power fields (× 400). **b** IgG4 immunostaining shows abundant IgG4-positive cells (× 400). **c** HE staining shows storiform fibrosis with spindle-shaped cells and inflammatory cells (× 100). **d**, **e** HE and Elastica van Gieson staining show obstructive phlebitis with infiltration of inflammatory cells obstructing the vein (× 100)

Regarding the histopathological findings based on the ICDC, lymphoplasmacytic infiltration, abundant IgG4-postive cells, SF, and OP were found by a Menghini-type needle in 12 (86%), 9 (64%), 5 (36%), and 1 (7%) patients, respectively (Table 2, Fig. 5). Consequently, 9 patients (64%) were histopathologically diagnosed with AIP (level 1 and 2 criteria of LPSP met by 5 [36%] and 4 [29%] patients, respectively). This histological finding contributed to a diagnosis of definitive type 1 AIP in a patient who had not been confirmed to have definitive type 1 AIP. On the other hand, conventional-type needle did not provide any diagnosis of AIP without detectable abundant-IgG4-postive cells, though lymphoplasmacytic infiltration was seen in only 29% of cases (4/14).

### Confirmed cytological diagnoses of no malignancy by EUS-FNA

The cytological diagnosis for distinguishing between malignant and benign cases was successfully confirmed in all patients treated with a Menghini-type needle and in 86% (12/14) of those treated with a conventional-type needle (not significant) (Table 2). The inability to make an accurate diagnosis was attributed to the acquisition of only a few cells showing degradation and the difficulty of performing a puncture due to the hardness of the pancreatic mass.

### Adverse events

Two patients (14%) treated with a Menghini-type needle with the rolling method had mild pancreatitis (Table 2). These patients had been punctured six times (transgastric and transuduodenal routes three times) in one and five times (transgastric route) in the other for the EUS procedure. Both patients had also undergone essential therapeutic-ERCP in a single session to achieve biliary drainage with pancreatographic imaging. These mild pancreatitis improved immediately with conservative therapy. No bleeding occurred in either group.

## Discussion

In this study, the 21-gauge Menghini-type biopsy needle with the rolling method was superior to the conventional 22-gauge FNA needles in terms of the histological diagnosis of AIP. We clearly demonstrated the reason was that the Menghini-type biopsy needle obtained remarkably large tissue by measurement of sample area of acquired tissue. Thus, definitive histological diagnosis was achieved in all 14 patients and the histological diagnosis of type 1 AIP was confirmed in 64% of them according to the ICDC, by EUS-FNB using the Menghini-type biopsy needle with the rolling method.

The ICDC published in 2011 do not recommend EUS-FNA or EUS-FNB for the histological diagnosis of AIP, as the findings are inconclusive [2]. However, developments in EUS-FNA technology have improved the EUS-FNB-based assessment of several pancreatic masses, including pancreatic cancer, pancreatic neuroendocrine tumors, and AIP. Previous published articles on the histological diagnosis for AIP by EUS-FNB were summarized in Table 3. The first of such reports described an EUS trucut biopsy with a 19-gauge trucut needle as a safe and accurate procedure for preserving the tissue architecture and aiding in the diagnosis of AIP [11, 12]. Also, a 19-gauge FNA needle provided a high rate of successful histological evaluations [14]. However, these needles are quite difficult to be handled under scope angulation and be punctured within the pancreatic mass of the head via the trasduodenal route [4, 11, 12, 26, 27], and an ex vivo study revealed that both 19-gauge needles induced greater resistance than 22- and 25-gauge needles for EUS-FNA [27], suggesting that these 19-gauge needles might be restricted to use by experts because of the handling difficulty.

**Table 3:** The histological diagnosis of AIP by EUS-FNB and EUS-FNA

References	Study design	Needle	Number of patients	Successful tissue acquisition for histological evaluation	Type 1 AIP						Type 2 AIP	
					Level 1	Level 2	Lympho-plasmacytic infiltration	IgG4-positive cells ≥ 10/HPF	Storiform fibrosis	Obliterative phlebitis	Level 1	Level 2
Levy [11]	Retrospective	19G Quick Core	3	100%	NM	NM	66%	NM	NM	NM	0	0
Mizuno [12]	Retrospective	19G Quick Core	14	100%	57%	21%	100%	64%	93%	36%	0	0
Imai [13]	Retrospective	22G NA-11 J-KB, 22G Echotip	21	100%	NM	NM	52%	0	0	0	0	0
Iwashita [14]	Retrospective	19G EchoTip	44	93%	NM	NM	52%	11%	86%	48%	0	0
Ishikawa [15]	Retrospective	22G EZ shot	47	91%	19%	11%	34%	35% (10/28^b^)	72%	0	0	6%
Kanno [16]	Retrospective	22G NA-11 J-KB, 22G EchoTip	25	80%^a^	56%	24%	92%	36%	80%	40%^c^	4%	0
Kanno [17]	Prospective	22G Expect	78	79%^e^	41%	17%	55%	24%	63%	49%	0	0
Morishima [18]	Prospective	22G Expect, 22G Expect slim, 22G EchoTip, 22G NA-11 J-KB	45	NM	0	60%	80%	60%	0	0	6%	2%
Cao [19]	Prospective	22G EchoTip ultra	27	93%^d^	19%	44%	67%	30%	67%	0	0	0
Bhattacharya [20]	Retrospective	22G SharkCore	2	100%	100%	0	100%	100%	100%	0	0	0
Kurita [21]	Prospective	22G Acquire	50	88%	56%	22%	84%	76%	56%	24%	0	0
		20G Procore	51	76%	26%	20%	60%	43%	25%	14%		
This article	Retrospective	21G Sonopsy	14	100%	36%	29%	86%	64%	36%	7%	0	0

*NM* no mention, *HPF* high-power field

^a^Samples with more than 10 high-power fields were considered adequate samples

^b^A total of 28 patients underwent immunostaining

^c^Including nine patients with suspected OP

^d^Samples with more than 5 high-power fields were considered adequate samples

^e^Samples with more than 1 high-power field were considered adequate samples

Regarding the use of 22-gauge FNA needles for histological evaluations for the diagnosis of AIP, three prospective studies have been already conducted. Kanno et al. reported that pancreatic tissues with more than 1 HPF were obtained in 80% (62/78) of patients, and AIP with an ICDC level of 1 or 2 was diagnosed in 58% (45/78) of the patients [17]. Cao et al. also reported that adequate tissue for histological evaluation was obtained in 93% (25/27), AIP with an ICDC level of 1 or 2 was diagnosed in 63% (17/27) of the patients and even head lesions were successfully punctured by a 22-gauge needle in the majority of patients (89%; 24/27) [19]. On the other hand, Morishima et al. reported that 22-gauge needles provided no patient with AIP with an ICDC level 1, and were not effective for the diagnosis of AIP due to the difficulty of obtaining pathological findings meeting three or more of the LPSP items [18]. A previous report comparing pancreatic biopsy specimens and surgically resected specimens showed that pancreatic specimens by core biopsy provided adequate histologic findings for AIP in only 22% of cases (2/9), whereas surgical specimens almost always provided sufficient findings for making a diagnosis [34]. In addition, the patchy distribution of specific histological findings, such as LPSP [35–37] and infiltration of IgG4-positive plasma cells [38] and the different severities [34] can affect the detection rate of histological findings. These data indicate that 22-gauge conventional FNA needles ensure relatively stable tissue collection but the amount of obtained specimen is not always sufficient for the histological diagnosis of AIP.

Recently, this 21-gauge Menghini-type biopsy needle was reported to be more useful for achieving the histopathological diagnosis of solid pancreatic masses than a standard 22-gauge needle [23, 24]. The most important feature of this needle is the equipment of an inner needle with plunger. Keeping the inner needle inside the outer needle during suction improves the ability to obtain adequate core tissue with preservation of a non-crushed tissue architecture. Indeed, this report revealed that the use of this needle was strongly associated with high cellularity (OR = 2.99, 95% CI 0.96–9.74, *P* = 0.062) and low blood contamination (OR = 28.63, 95% CI 6.67–162.44, *P* < 0.001) in a histological evaluation [23]. Remarkably, those good-quality tissue with less blood contamination was also achieved in our cases, as shown in Fig. 3. We also consider that the unique approach of making only three passes with rotation of the needle in the lesion via the “rolling method” contributed to the acquisition of wrenched core tissue with relatively little blood contamination, as with the liver biopsy technique using the Majima needle [25]. This method is based on the notion that a large amount of cored, good-quality tissue with little contamination may be able to be acquired by reducing the number of strokes as much as possible. In addition, even the transduodenal approach resulted in sufficient tissue collection in all 16 patients [23] as well as in 1 patient included in our study (100%), despite some rigidity. Taken together, these results strongly support the notion that this 21-gauge Menghini-type biopsy needle with the rolling method can be a reliable way of obtaining abundant tissue with preserved architecture for a histological evaluation in order to make a diagnosis of AIP.

Another important role of EUS-FNA or EUS-FNB for patients with suspected AIP is the need to rule out the presence of malignancy. The accuracy of the cytological evaluation has recently dramatically improved due to the advent of rapid onsite evaluation (ROSE) as well as the development of various needles and puncture techniques, such as the fanning technique [39] and slow-pull technique [40]. In our study, cytological evaluations were consistently successful using both types of needles (93%: 26/28). On the contrary, confirming adequate tissue acquisition for histological evaluations during EUS-FNB is usually difficult, because the obtained sample contains mucus, blood contamination, adipose tissue as well as required pancreatic tissue. As one of solution for overcoming this issue, the identification of a macroscopic visible core which consisted of whitish or yellowish pieces contributed to a high acquisition rate of the histological core with a success rate of 87% (187/216) for all passes in the MOSE study [32]. Thus, the 21-gauge Menghini-type needle combined with the MOSE method can be a powerful tool for confirming the histological diagnosis of AIP.

Two patients treated with a Menghini-type needle developed pancreatitis after the procedure. We generally performed EUS-FNB and subsequent ERCP in a single session, based on our previous findings, which demonstrated the efficacy and safety of single-session EUS-FNB and ERCP [29–31]. Minaga et al. reported that no adverse event occurred in 47 patients with pancreatic masses treated with the Menghini-type needle [23]. On the other hand, Naito et al. reported that the incidence of post-ERCP pancreatitis was slightly lower in type 1 AIP than non-AIP cohort (1.2% vs. 5.8%, *P* = 0.119) [41]. The relation between this needle and pancreatitis cannot be referred to in this study, so further assessment is needed with more cases. Theoretically, the needle simply rotates in the pancreas using the additional “rolling method”. Therefore, bleeding can be considered an alarming risk associated with this method, especially if adequate core tissue is acquired from pancreas. Fortunately, no bleeding occurred in any of our cases. However, further assessments in more cases will be needed to confirm the safety.

On reviewing the previous reports described in Table 3, the utility of some FNB needles, such as the Franseen type (Acquire) and fork-tip type needles (SharkCore), has recently been reported for the diagnosis of AIP. These needles seemed to contribute to a good histological assessment for diagnosing AIP. Interestingly, the Menghini-type needle with the rolling method is unlikely to be inferior to these FNB needles. In addition, one specific merit of this procedure is that even a few strokes can provide a substantial amount of good-quality tissue. This merit will prove particularly beneficial for lesions that are not easy to puncture due to the scope position, helping to improve the diagnostic ability. However, which needle is the best remains unclear, and a randomized control study will be needed to clarify this point.

Several limitations associated with the present study warrant mention. First, this was a retrospective, single-center study with a small number of enrolled patients. However, the amount of specimen acquired by the Menghini-type needle with the rolling method was quite large, as shown in Figs. 2 and 3. In addition, in the two patients treated with both needles, a substantial amount of tissue was acquired by the Menghini-type needle with the rolling method, while relatively little tissue had been acquired with the previously used conventional-type needle, as shown in Fig. 4. Second, we evaluated patients treated with 22-gauge conventional-type needles as historical controls, and the needles were selected based on the two experts' preference. In addition, these patients were characterized by segmental or focal enlargement of the pancreas, the adoption of a transduodenal approach for EUS, and a low number of punctures, which may have resulted in worse outcomes for these patients than for others in the present and previous studies. However, even though several prospective studies conducted in academic institutions with expert pathologists showed excellent outcomes using 22-gauge FNA needles for the diagnosis of AIP [17–19, 21], the diagnostic rate, proportion of Level 1 or 2 in ICDC and positive rates of specific histological findings differed markedly between these studies. In reality, the biopsy-based diagnosis of AIP is challenging and remains unstandardized. Just recently, Notohara et al. advocated guidance for diagnosing AIP with biopsy tissue in order to standardize pathology reports of pancreatic biopsies for diagnosing type 1 AIP [42]. These differences in results are also consistent with a previous study, wherein small samples obtained by EUS-FNA induced low diagnostic agreement among pathologists from not only academic institutions but also non-academic institutions [43]. Although the acquired sample area has not been evaluated in the prospective studies, we believe that their superb outcomes are not necessarily adapted in all institutions around the world. Third, the patients in each group underwent different biopsy techniques, whether the rolling method or the standard aspiration technique. This means that this study was not a pure comparison of both needles. We emphasize that the utility of the 21-gauge Menghini-type needle combined with the rolling method was superior to that of the conventional 22-gauge needle with the standard aspiration technique in terms of the amount of acquired tissue. However, we cannot state whether the needle or technique had a greater effect on the amount of tissue acquired in this study. To clarify this point, a randomized control study comparing the 21-gauge Menghini-type needle with and without the rolling method will need to be conducted. Fourth, all patients were type 1 AIP, so the performance of the Menghini-type needle for the diagnosis of type 2 AIP remains unclear.

Despite these limitations, however, our study demonstrated that the outcomes in patients treated with the Menghini-type needle with the rolling method were undoubtedly preferable to the outcomes in patients treated with other approaches, as proven by the objective evaluation of the acquired tissue area. This superiority is attributed to the ability to acquire an abundant amount of tissue, which was exactly important for obtaining a histological diagnosis of AIP [22]. In addition, we emphasize that histology of larger sample would contribute to strengthened diagnostic performance and agreement among pathologists of varying expertise. However, further study is needed to be validated in a large number of AIP patients.

In conclusion, the 21-gauge Menghini-type biopsy needle with the rolling method is useful for the EUS-guided histopathological diagnosis of AIP due to its abundant acquisition of good-quality tissue from the pancreas. It will significantly aid in the definitive diagnosis of AIP.

## Data Availability

The datasets used and/or analyzed during the current study are available from the corresponding author on reasonable request.

## Abbreviations

AIP: Autoimmune pancreatitis
ASGE: American Society of Gastrointestinal Endoscopy
EUS-FNA: Endoscopic ultrasound-guided fine-needle aspiration
EUS-FNB: Endoscopic ultrasound-guided fine-needle biopsy
GEL: Granulocytic infiltration of duct wall
HPF: High-power field
ICDC: International Consensus Diagnostic Criteria
IDCP: Idiopathic duct-centric pancreatitis
IQR: Interquartile range
LPSP: Lymphoplasmacytic sclerosing pancreatitis
MOSE: Macroscopic on-site quality evaluation
OP: Obliterative phlebitis
ROSE: Rapid onsite evaluation
SF: Storiform fibrosis
